# Locked-in syndrome after basilary artery thrombosis by mucormycosis masquerading as meningoencephalitis in a lymphoma patient

**DOI:** 10.3332/ecancer.2013.382

**Published:** 2013-12-19

**Authors:** Fausto Maffini, Emilia Cocorocchio, Giancarlo Pruneri, Guido Bonomo, Fedro Peccatori, Laura Chiapparini, Silvia Di Vincenzo, Giovanni Martinelli, Giuseppe Viale

**Affiliations:** 1 Division of Pathology, European Institute of Oncology, Milan 20141, Italy; 2 Division of Haematoncology, European Institute of Oncology, Milan 20141, Italy; 3 Division of Radiology, European Institute of Oncology, Milan 20141, Italy; 4 University of Milan School of Medicine, Milan 20141, Italy

**Keywords:** mycosis, mucorales fungi, stroke, thrombosis, cutaneous lymphomas, oncology, infectious disease

## Abstract

Locked-in syndrome is a rare clinical syndrome due to basilary artery thrombosis generally associated with trauma, vascular, or cardiac malformation. It can present as various types of clinical evolution and occasionally masquerades as other pathological conditions, such as infective meningoencephalitis. These complications are the cause of diagnostic delay, if not promptly recognised, followed by patient death. We report the case of a 42-year-old female with a systemic B and cutaneous T-cell non-Hodgkin’s lymphoma, with a severe neutropenia lasting over a year, who eventually developed a rapid and fatal fungal mucormycosis sepsis following a skin infection on her right arm, associated with locked-in syndrome and meningoencephalitis.

## Introduction

Locked-in syndrome (LIS) is a clinical condition in which patients are conscious but unable to move or speak. It is often secondary to ventral pontine lesions, although LIS can be observed in patients after trauma, vascular, or cardiac malformation [1–3]. Diagnosis is sometimes difficult because, in the acute and chronic phase, LIS can simulate a comatose or vegetative status. Mucormycosis is an opportunistic disease caused by the glomeromycota class of fungi [4], able to induce potentially fatal infections in immunocompromised patients. In recent years, its incidence in several countries seems to have increased [5, 6]. Diabetes, hyperglycaemia, long-lasting neutropenia, steroids, iron overload, and iron chelation with deferoxamine are the most well-known risk factors [7].

We describe the clinical case of a patient affected by non-Hodgkin’s lymphoma (NHL), who developed a fatal meningoencephalitis associated to LIS due to mucormycosis sepsis.

## Clinical case

A 42-year-old woman came to our attention for relapsed NHL. Initial staging revealed a G2 stage IIIBX centrofollicular NHL. The patient previously received several therapy lines, such as a fludarabine-based regimen, chlorambucil, vincristine, and prednisone combination, steroids, and rituximab, developing neutropenia and thrombocytopaenia. Two months after the completion of immunotherapy, she developed multiple erythematous nodular skin lesions, which were biopsied, revealing the presence of panniculitis-like T-cell NHL, confirmed by the presence of a T-cell receptor rearrangement by polymerase chain reaction (PCR). She rapidly progressed despite the administration of CHOP (cyclophosphamide, vincristine, adriamycin, and prednisone) chemotherapy regimen. Thus, even though the persistent pancytopaenia, a dose-adjusted ESHAP (Aracytin, methylprednisolone, cis-platinum) regimen was started (day 0). A concomitant broad-spectrum anti-infective therapy with ceftazidime, amikacin, teicoplanin, fluconazole, and acyclovir was administered because of a previous febrile episode classified as a fever of unknown origin. On day 10, during deep neutropenic phase, while the lymphoma skin lesions were shrinking, a single papule was observed on the patient’s right arm. Two days later, the lesions became painful and developed into a black eschar with induration, oedema, and erythema, reaching a diameter of 45 mm. A cytological smear for culture examination was negative.

On day 15, the patient became febrile, while chest x-rays and blood cultures were still negative. Ceftazidime, amikacin, fluconazole, and acyclovir were discontinued and substituted by meropenem 6000 mg/daily, liposomal amphotericin B 1.5 mg/kg/daily, and foscarnet 180 mg/kg/daily.

**Figure 1.**
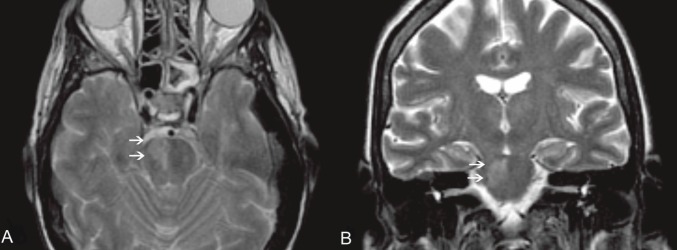
An MRI of the brain, T2 W images, axial (A) and coronal view (B). Clearly visible in the right portion of the pons is a hyperintense area (white arrows), according to pathologic findings (mucormycosis localisation).

On day 17, the patient developed areflexia, paresis, slurred speech, and lateral lingual swelling; a brain computed tomography (CT) scan was negative; brain magnetic resonance images (MRIs) showed an alterated signal in T2 sequences on the pontobulbar zone (Figure 1a,b). A lumbar puncture was performed showing haemorrhagic cerebrospinal fluid with a granulocyte count of 60/mmc, while culture and chemical examinations were negative. A punch biopsy of the skin lesion was performed and *Mucor *spp. was identified. The patient’s neurological symptoms rapidly worsened, with appearance of positive bilateral Babinski, coma, and decerebration. Meropenem and teicoplanin were discontinued; ceftriaxone 4000 mg/daily and gentamicin 300 mg/daily were introduced due to meningoencephalitis.

On day 20, due to persistent neurological symptoms, an MRI was performed, showing an increase in the altered signal in T2 sequences without enhancement after gadolinium on the pontobulbar zone with mesencephalon and thalamus involvement, suggestive of an ischaemic lesion. A further lumbar puncture still showed haemorrhagic cerebrospinal fluid, with a granulocyte count of 730/mmc and negative culture and chemical examination. The cytology showed only arachnoid cells intermingled with amorphous background material associated with an extensive granulocyte nest.

On day 21, blood count showed leukocytes and neutrophils rescue; LNH skin lesions were almost completely regressed, but the patient experienced polyuria, hypernatremia, and hyperchloremia as in the case of an inappropriate anti-diuretic hormone secretion syndrome. The symptoms regressed with hydration and desmopressin administration. On day 23, the patient died.

An autopsy was subsequently performed. The skin lesion appeared to be like an eschar with irregular ulcerations of borders. The gross brain analysis showed the presence of extensive oedemas and white-creamy purulent material involving the leptomeningeal brainstem, mesencephalon, pons, and bulbus area. The pial vessels were engulfed by blood, and the Willis heptagons showed an induration around the basilar artery. The brain was soft, and the grey matter was not discernible.

**Figure 2.**
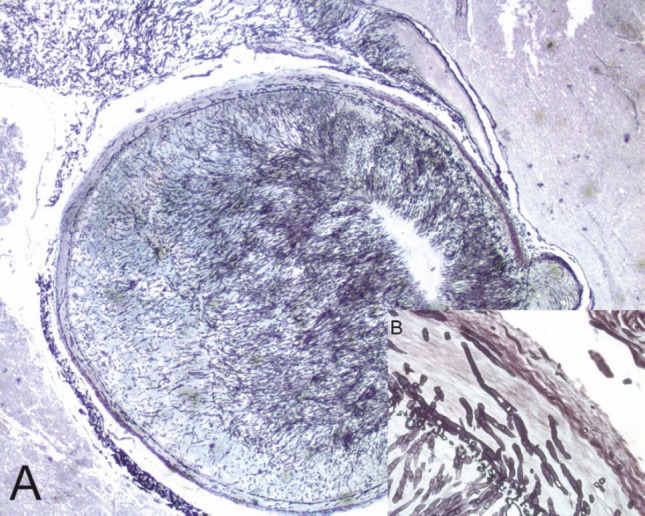
A mycotic thrombus occluding the lumen of the basilar artery (A) (2x original magnification, Grocott staining). The fungi showed typical broad, haphazardly branched hyphae that performed a basilary artery wall, inset (B) (40x original magnification, Grocott staining).

**Figure 3.**
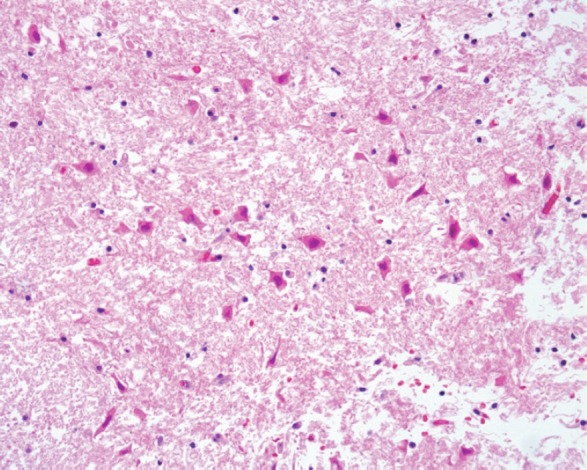
The cerebral parenchyma showed red neurons typically observed in early ischaemic damage (20x original magnification HE staining).

The histological evaluation showed a mucor thrombus of the basilary artery associated with a wall perforation, leading to subsequent meningeal and parenchymal involvements. The Grocott stain showed no septate hyphae, a lack of any dycotomic subdivision with irregular borders and insertion angle. All these features are pathognomonic of *Mucor *spp. infection (Figure 2a,b). The brain parenchyma showed an early ischaemic damage (red neurons) in the presence of to complete neuronal loss areas, microglial activation, oedema, and vascular engulfment (Figure 3). Thus, the death was attributed to infective meningoencephalitis associated with basilary artery thrombosis. Interestingly, a complete regression of the subcutaneous neoplastic infiltrate was documented by morphological, phenotypical, and genotypical (absence of a T-cell receptor rearrangement) analyses. The bone marrow was markedly hypocellular with a normal maturation of the haematopoietic cell lines without evidence of disease.

## Discussion

This case confirms the aggressive behaviour of this disease. The infection started atypically. In fact, primary cutaneous manifestation is typical of immunocompetent patients (usually after trauma, like a burn), while in immunocompromised patients, the rhino-orbital-cerebral disease seems to be the most frequent manifestation [8]. From the appearance of the skin lesion, clinical conditions quickly worsened in two days and exitus arose after less than two weeks from the appearance of the skin lesion. Even if an MRI identified a wide cerebral ischaemic area and the cerebrospinal fluid showed infective meningitis, the pathogen agent was not immediately recognised. The delay in diagnosis was due to the negativity of the first cultural exams that were performed on material withdrawn from the central part of the lesion and on the cerebrospinal fluid. Also the concomitant pancytopaenia, due to the recent chemotherapy, made all invasive diagnostic and therapeutic procedures difficult to continue with. However, an active antifungal therapy with liposomal amphotericin B was empirically promptly started. The subsequent cutaneous biopsy performed on the board of the skin lesion allowed the fungal identification, but at that time, the patient was already in a comatose state and, despite the haematological recovery, surgery was not considered.

Basilar artery thrombosis due to *Mucor *infection is rarely observed and reported [9]. It is a consequence of fungal iphae vascular wall tropism that can cause thrombus formation, vascular obstruction, and/or perforation [10]. With early blood diffusion and multiorgan involvement [10, 11], the first clinical manifestation could be an LIS with tetraplegia, areflexia, blurred vision, and dysartria [1–3]. Physically, this syndrome could masquerade as concomitant meningoencephalitis with fever, headache, and stiffness of the neck. This condition should be promptly recognised, because it rapidly worsens into coma and death [1–3]. 

The availability of new antifungal drugs has improved outcomes, but the mortality rate still remains high, mainly in the haematological setting, where 91% of cases occur [8]. In neutropenic patients, ante-mortem diagnosis is uncommon due to rare positivity of blood cultures [12]. Conversely, iphae and spores are present in the first site of involvement, and a diagnosis can be quickly made by a simple biopsy or scraping in the lesion board or with cytological methods [12]. Other diagnostic tools such as quantitative PCR are still under evaluation [13, 14]. Imaging could help in early diagnosis; while MRI is sensitive enough to detect infection for CNS involvement, a CT scan allows detection of lung infections. Therapeutic strategy of mucormycosis infection is complex and often requires the combined use of surgery and systemic therapy. Early identification of infection and correction of underlying disease is crucial for successful treatment. In fact, the delay in starting specific therapy for more than 6 days is associated with a higher mortality rate at 12 weeks [15]. Elective antifungal drugs are polyenes, such as amphotericin B deoxycholate, and its lipid formulations. Liposomal amphotericin B seems to achieve a higher resolution rate and mortality rate reduction with respect to amphotericin B deoxycholate. Furthermore, the lipid formulation allows a better diffusion on CNS and a renal toxicity reduction [16–18]. Triazoles also constitute a possible choice. Posaconazole was successfully used as salvage therapy, alone or in combination with amphotericin B [19], although data on its use as first-line therapy are not recommended [20, 21].

Echinocandins seems not to be effective on mucorales as a monotherapy [22], although activity on *Rhizopus oryzae *is reported with caspofungin and liposomal amphotericin B combination [23].

Several other agents were investigated, like deferasirox, an Iron chelant agent that has an antifungal effect, synergistic with liposomial amphotericin B [24] or immunosuppressive agents like tacrolimus that could synergistically act with posaconazole or caspofungin [25].

## Conclusion

Mucor infection should always be considered in the differential diagnosis of systemic infection in immunocompromised hosts, and all skin lesions should be evaluated to ensure correct and prompt treatment.
